# Formulation, In Vitro Characterization and Antibacterial Activity of Chitosan-Decorated Cream Containing Bacitracin for Topical Delivery

**DOI:** 10.3390/antibiotics11091151

**Published:** 2022-08-25

**Authors:** Rumana Zaib Khattak, Asif Nawaz, Maha Abdallah Alnuwaiser, Muhammad Shahid Latif, Sheikh Abdur Rashid, Asghar Ali Khan, Soha A. Alamoudi

**Affiliations:** 1 Advanced Drug Delivery Lab, Gomal Centre of Pharmaceutical Sciences, Faculty of Pharmacy, Gomal University, Dera Ismail Khan 29050, Pakistan; 2 Department of Chemistry, College of Science, Princess Nourah bint Abdulrahman University, P.O. Box 84428, Riyadh 11671, Saudi Arabia; 3 Department of Agronomy, Faculty of Agriculture, Gomal University, Dera Ismail Khan 29050, Pakistan; 4 Biological Sciences Department, College of Science and Arts, King Abdulaziz University, Rabigh 21911, Saudi Arabia

**Keywords:** bacitracin, cream, antibacterial activity, in vitro evaluation, chitosan

## Abstract

(1) Background: Bacitracin is a broad spectrum antibiotic that is used against various microorganisms. Chitosan is a natural polymer that has been widely investigated as an antimicrobial agent for preventing and treating infections owing to its intrinsic antimicrobial properties, as well as its ability to effectively deliver extrinsic antimicrobial compounds to infected areas. Topical drug delivery offers important benefits for improving the therapeutic effect and reducing systemic side effects of administered compounds/drugs. The topical use of chitosan-decorated bacitracin-loaded cream improves the permeation of the drug across the skin and enhances the drug bioavailability by prolonging the residence time of the drug when applied topically, as well as producing synergistic effects and reducing the side effects of the drug. Topical chitosan-decorated cream can be a promising approach to administer the drug more efficiently and enhance the efficacy of treatment in wound healing and antibacterial activity. (2) Methods: This study was conducted to prepare, assess and investigate the synergistic antibacterial activity of a chitosan-coated bacitracin cream. The results were compared to the antibacterial activity of simple bacitracin-loaded cream. The prepared cream was evaluated for various in vitro characteristics such as rheology, pH, viscosity, drug content and antibacterial activity studies. (3) Result: The formulations were found to be stable regarding color, liquefaction and phase separation at all accelerated conditions. It was observed that with time, substantial variations in the pH of the preparations were found. The introduction of chitosan results in controlled release of the drug from the formulations. The antibacterial activity of the formulated creams was assessed with the disc diffusion method against *Staphylococcus aureus* *(ATCC),* *Escherichia* *coli (STCC),* *Pseudomonas aeruginosa* *(ATCC)* and *Bacillus cereus* *(ATCC)*. The strains, *E. coli*, *S. aureus*, *P. aeruginosa* and *B. cereus* were susceptible to 50 µg chitosan-decorated bacitracin cream, showing inhibition zones of 10 ± 0.6, 34 ± 1.5, 31 ± 0.76 and 21 ± 2.02 mm, respectively. The zones of inhibition for simple bacitracin-loaded cream were significantly smaller than chitosan-decorated cream, at 2 ± 0.2, 28 ± 0.92, 15 ± 0.5 and 11 ± 1.25 mm (ANOVA; *p* < 0.05), respectively. (4) Conclusion: It was observed that the zones of inhibition of simple bacitracin-loaded cream were significantly smaller than those of chitosan-decorated bacitracin-loaded cream. Chitosan synergistically improves the antimicrobial activity of bacitracin. Hence, the developed formulation was effective and should be considered as a suitable candidate for topical management of skin infections and wound healing.

## 1. Introduction

Chitosan is a derivative of chitin. It is polysaccharide with molecular weight between 300 to 1000 kDa [1]. It is the second most commonly used natural polymer after cellulose [2]. The chemical structure of chitin is 1-4 linked 2-acetamido-2-deoxy- β-D-glucopyranose [3]. Since the 1970s, chitosan has been popular in science and industry due to its specific structure, chemical composition, compatibility, biodegradability and other intrinsic properties [4].

Chitosan has many therapeutic qualities such as antibacterial, antifungal, healing property, anti-cholesteric properties, anticancer ability, and an immune-system enhancing effect. The antibacterial activity of chitosan has been observed to be enhanced if its degree of deacetylation and molecular weight is increased, and vice versa [5]. Chitosan’s antimicrobial properties, as well as its modes of action, demonstrate that it is a versatile substance with a wide range of therapeutic applications. Chitosan is also used as a wound-healing accelerator in medicine due to its property of enhancing the functions of inflammatory cells. Chitosan appears to have no adverse effects after implantation in tissues and, for this reason, it has been used for a wide range of biomedical applications [6]. Chitosan is used to inhibit fibroplasia in wound healing and to promote tissue growth and differentiation in culture. As a natural polymer, chitosan has been widely investigated as an antimicrobial agent for preventing and treating infections owing to its intrinsic antimicrobial properties, as well as its ability to effectively deliver extrinsic antimicrobial compounds into infected areas. Keeping in view all these benefits, chitosan has been added to topical formulations (cream) and is considered to be a good candidate for burn and wound management [7].Topical drug delivery offers important benefits for improving the therapeutic effect and reducing systemic side effects of the administered compounds/drugs [8]. The topical application of drugs using particulate systems consisting of chitosan is one of the most popular drug delivery routes. The aim of the topical use of chitosan particles is to improve the permeation of drug across the skin or enhance drug bioavailability by prolonging the residence time of drugs applied topically or to produce synergistic effects, as well as to reduce the side effects of the drugs [9].

Bacitracin is a topical antibiotic used to treat skin injuries such as cuts, scrapes and burns by medical personnel and the general population [10]. The United States FDA permitted the clinical use of bacitracin in 1948 for the cure and prevention of both acute and chronic skin infections. Intramuscular injection of bacitracin can also be given for the systemic treatment of staphylococcal pneumonia and emphysema. However, in 2020, the FDA requested the withdrawal of bacitracin for injections from the market [11]. To date, there is no evidence of any non-FDA permitted uses of topical bacitracin. Bacitracin is a combination of many closely related cyclic polypeptide antibiotics that have both bacteriostatic and bactericidal effects. Gram-positive bacteria such as Staphylococcus spp, Corynebacterium spp, Streptococcus spp, Actinomyces spp and Clostridium spp are susceptible to bacitracin. Gram-negative bacteria such as Neisseria spp. also possess susceptibility, while many gram-negative bacteria are resistant [12]. Bacitracin interferes with the dephosphorylation of C55-isoprenyl pyrophosphate and bactoprenol pyrophosphate. Both of these lipid’s function as membrane carrier molecules that transport the building-blocks of the peptidoglycan bacterial cell wall outside of the inner membrane; thus, their dephosphorylation leads to membrane damage. Topical bacitracin prevents the transfer of mucopeptides into the cell walls of bacteria and is absorbed through burnt or granulated skin. Bacitracin inhibits the growth of microorganism by causing leaks in the cell wall of bacteria via the ionic surface interaction method, preventing protein synthesis and the formation of mRNA [13].

The need to develop new effective antimicrobial agents has become imperative due to the rapid development of antibiotic resistance. New synthetic compounds require long duration. Rapid way to overcome this problem is the use of natural compounds including natural polymers such as chitosan. Natural compounds play a key role as sources of new scaffolds for antibiotics. Wounds are a serious health issue all around the world. Wounds can cause major consequences as a result of subsequent microbial infections [14]. Simple cream formulations have low stability, drug entrapment efficacy and penetration rate. To overcome all these problems topical chitosan-decorated cream can be a promising approach to administer the drug more efficiently and enhance the efficacy of treatment in skin infections such as wound healing. The aim of the present study was to develop chitosan-decorated bacitracin-based cream for topical delivery.

## 2. Materials and Methods

### 2.1. Materials

The model drug used was bacitracin (Sigma-Aldrich, St. Louis, MO, USA). Chitosan (molecular weight; 15,000 Da and degree of deacetylation; 85%; Sigma-Aldrich, USA) of low molecular weight was used in this study. Liquid paraffin and white soft paraffin (The Dow Chemical Company., Washington, MD, USA) were used in the preparation of cream. Cetyl alcohol and beeswax (Sigma-Aldrich, St. Louis, MO, USA,) was used in preparation of the oil phase of the cream. All the chemicals used in this study were of analytical grade.

### 2.2. ATR-FTIR Analysis

The FTIR analysis was performed using an ATR-FTIR spectrometer (L1600300, PerkinElmer, Beacons field, MA, USA. The FTIR spectra of bacitracin, chitosan and formulations C1 and C2 were obtained. The recording range of the spectrum was 600–4000 cm^−1^ at 32 scans per minute with a resolution of 4 cm^−1^ in transmission mode. The samples were analyzed without further processing and directly placed on zinc selenide. Three spectra of each sample were taken and results were averaged.

### 2.3. Preparation of Base Cream

Chitosan-decorated bacitracin cream for topical application was prepared in two steps/phases, i.e., aqueous and oil phases. For the aqueous phase, 19 g of distilled water was taken in a 50 mL beaker and placed on a hot plate magnetic stirrer at 40 °C. 1 g of bacitracin was taken and dissolved in distilled water. Chitosan (1 g) was accurately weighed and added to the drug solution, then stirred at 500 rpm until completely dissolved. For the oil phase, 20 g liquid paraffin, 37 g white soft paraffin, 4 g cetyl alcohol and 18 g beeswax were accurately weighed with the help of a digital balance and placed in separate beakers. All the beakers were wrapped in aluminum foil and placed in a water bath at 70 °C for 30 min. Cetyl alcohol was placed on a hot plate magnetic stirrer and all the chemicals were added to it with continuous stirring at 500 rpm and temperature at 40 °C.

For the preparation of cream, the oil phase was placed on a hot plate magnetic stirrer at 40 °C and 500 rpm. The aqueous phase was gently added to the oil phase, with continuous stirring for 30 min at 500 rpm until the aqueous phase was completely dissolved. The prepared cream was taken from the hot plate magnetic stirrer and moved to a suitable container for cooling at room temperature. The prepared cream was then evaluated for various physical characteristics (see composition in Table 1).

### 2.4. In Vitro Characterization of Cream

#### 2.4.1. Physical Appearance

The physical appearance of the prepared creams was observed in terms of color and roughness, and was graded.

#### 2.4.2. Determination of pH

The formulated cream encoded as C1 and C2 were checked by digital pH meter (InoLab^®^, Xylem Analytics, Dr. Karl Slevogt Street 1. 82362 Weilheim, Germany) for the determination of pH. The pH was measured at different temperatures (8 °C, 25 °C, 40 °C, and 40 °C + 75% RH) during the study duration of 28 days. The pH values were calculated in triplicate and averaged as mean ± SD [15].

#### 2.4.3. Homogeneity, Organoleptic and Smear Tests

To determine the composition of the prepared creams, a homogeneity test was performed. This test revealed the homogeneity of the prepared formulations by physical touch. A smear test was performed to check the run-off effect and nature of the cream. The test was performed by applying a small amount of cream on the skin to check if it was greasy or non- greasy in nature [16].

#### 2.4.4. Viscosity

The viscosity of prepared creams were checked with the help of a digital viscometer (AMETEK Brookfield, 11 Commerce Blvd, Middleboro, MA 02346, United States) over the study period of 28 days at different temperatures (8 °C, 25 °C, 40 °C and 40 °C-RH). Spindle no. 2 was used for the determination of viscosity at 6, 12, 30 and 60 rpm. The result was computed in centipoise. All the reading were taken in triplicate and averaged as mean ±SD [17].

#### 2.4.5. Spreadability

To test the spreadability of the prepared creams, the slip and drag method (parallel plate method) was utilized. In this technique, one glass slide is attached to a wooden block and a second one is put above the first one with similar dimensions. One gram of cream was poured on the fixed slide and distributed using a spreader. Cream was squeeze in between the slides. A 100 g solid support was placed on the glass slides to uniformly spread cream over the slide, and the top slide was freely moved under a 10 g weight linked to the upper slide’s hook. The time it took for the top slide to glide over the bottom slide was recorded, and spreadability was computed in g.cm/s. The experiment was performed for all of the cream formulations [15].

#### 2.4.6. Drug Content

Drug content was measured by taking 1 g of cream (C1 and C2) in a 100 mL flask containing distilled water. The conical flask was stirred continuously for 30 min until a clear solution was obtained. The obtained solution was filtered through Whatman filter paper (grade 42) and the filtrate was collected. To make dilutions, flasks were taken with 10 mL distilled water and 1 mL filtrate was added from the 100 mL solution. Absorbance of the resulting dilutions was checked at 215 nm by using a UV-spectrophotometer, and the content of bacitracin was calculated by using the following equation [18]:

% Drug Content = (Absorbance of sample/absorbance of standard) × 100%(1)

#### 2.4.7. Stability Studies

These studies was done to determine the change in color, liquefaction and phase separation. The formulations coded as C1 and C2 were divided into four samples, each of which was stored at different temperatures, including 8 °C, 25 °C, 40 °C, and 40 °C, ±75% RH. The study was conducted for 60 days. Samples were thoroughly monitored with regards to color change, liquefaction and phase separation under various storage settings [19].

### 2.5. In Vitro Drug Release

The drug release pattern was observed by using a Franz diffusion cell (PermeGear, Hellertown, PA, USA). The hydrophilic cellulose acetate membrane (ADVANTEC C300A142C) was submerged in acetate buffer solution (pH 5.5; simulating skin pH) and inserted between the receptor and donor compartments of the Franz diffusion cell. One gram of cream was added to the donor compartment of the Franz diffusion cell while the cell was continuously stirred using a magnetic bar at 100 rpm. At predetermined time intervals (0, 0.5, 1, 1.5, 2, 4, 8, 12, 16, 20 and 24 h), 2 mL of samples were taken from the receptor compartment using a syringe and examined on a UV visible spectrophotometer at 215 nm for quantification of bacitracin [20].

### 2.6. Antibacterial Assay

Antibacterial activity assay of the formulations (C1 and C2) was performed against the following species: *Staphylococcus aureus*, *Pseudomonas aeruginosa, Escherichia coli* and *Bacillus cereus*.

#### 2.6.1. Preparation of Nutrient Agar Media

The nutrient agar media was made by adding 0.5% peptone, 0.3% beef extract, 1.5% agar and 0.5% NaCl in 1 L distilled water. The complete dissolution of all ingredients was assured by continuous stirring and heat. The medium was autoclaved at 121 °C for 15 min and the solution was allowed to cool at room temperature. The agar media was taken carefully and poured in sterile petri dishes until it set on the surface; then, it was refrigerated by replacing the lid of the petri dish. Nutrient agar media was used for plating bacterial species for liquid overnight culture [21].

#### 2.6.2. Disc Plate Method

The test was carried out using the disc plate diffusion method. The microorganisms were sub-cultured (the previous day) to ensure that the bacteria were in the log phase of growth. The bacterial inoculum was spread on the surface of a nutrient agar plate containing 25 mL of media with a sterile cotton swab. The dried inoculated plates were impregnated with sterile 5 mm paper discs (Difco). Petri plates were incubated for 24 h at a temperature of 37 °C. Within 15 min of inoculation, the samples were placed in the plates. The test was performed using 50 µg of chitosan-decorated bacitracin cream (C2) per disc as a microbiological susceptibility control, and the same procedure was repeated for non-chitosan-decorated bacitracin cream (C1). The inoculation plates were incubated at 37 °C for 24, 48 and 72 h before being checked for inhibition zones measured in mm using a ruler for each disc displaying inhibition zones [22]. The results of the chitosan-decorated bacitracin cream was compared with those of the non-chitosan-decorated bacitracin cream.

### 2.7. Statistical Analysis

All the experiments were conducted in triplicates and the results were averaged. The data were evaluated by using one-way analysis of variance (ANOVA) using SPSS. Post hoc multiple comparisons were applied when necessary. A *p* value of <0.05 was considered significant.

## 3. Results and Discussion

### 3.1. ATR-FTIR Analysis

**Figure 1:**
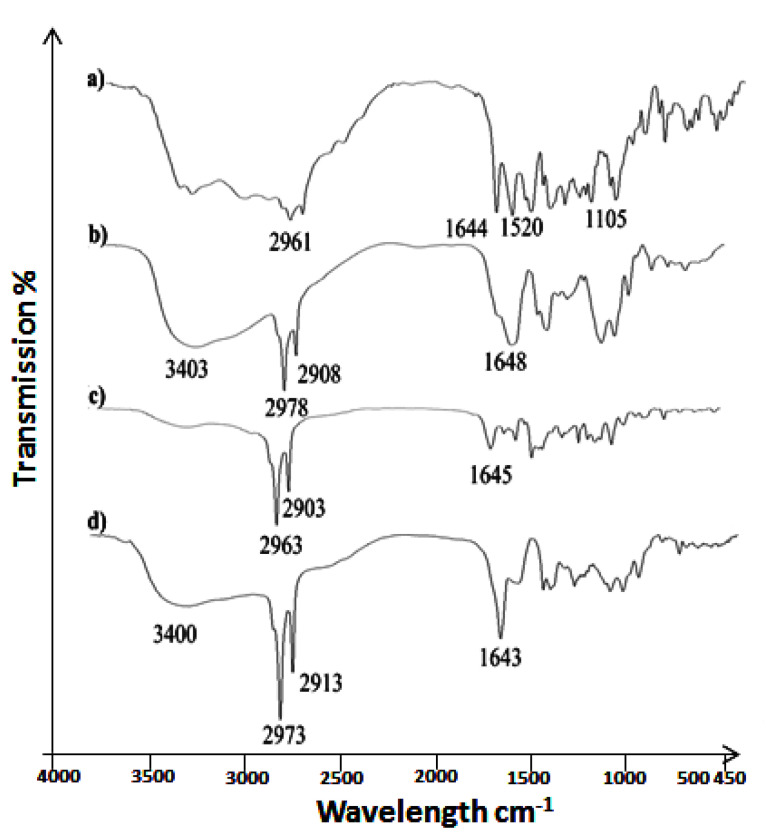
FTIR spectra of (**a**) drug, (**b**) chitosan, (**c**) Formulation C1 and (**d**) Formulation C2.

The characteristic peaks of the drug (bacitracin) were 2961 cm^−1^ (stretching vibrations of C-H and C-C), 1644 cm^−1^ (stretching vibrations of C-O), 1520 cm^−1^ (stretching vibrations of C-H and C-C) and 1105 cm^−1^ (C-O alcohol bond). Chitosan’s characteristic peaks were at 3403 cm^−1^ representing NH functional groups (primary amine). The absorption band at around 2978 cm^−1^ can be attributed to CH symmetric stretching and 1644 cm^−1^ can be attributed to stretching vibrations of C-O. The FTIR spectra of Formulation C1 contains the characteristic peaks of the drug, showing that the drug is free in the cream base. The characteristic peaks of the drug were weakened in Formulation C2, as shown in Figure 1d, and the chitosan characteristic peaks were dominant in Formulation C2, showing the successful coating of the drug with chitosan (Figure 1).

### 3.2. Physicochemical Characterization of Cream

#### 3.2.1. Physical Appearance

The prepared cream was organoleptically evaluated for its color, which was observed as off white to light yellow (C1 and C2). The prepared cream had a homogeneous composition with semisolid consistency. The cream also possessed good moisturizing properties with a pleasant odor. The prepared cream showed no phase separation, passed all the physical tests and was considered best candidate to be used topically.

#### 3.2.2. Determination of pH

The pH of cream is a significant factor to determine its efficiency. Ijaz et al. stated that the pH value of the prepared formulation must be in the range suitable for skin [15]. The pH of the formulation was adjusted to the normal range by adding NaOH dropwise under continuous stirring. The pH of the formulation in the current study was in the range of 4.5 to 6.0, as shown in Table 2, which was within the official limit and in accordance with the pH of the skin. The same phenomena were discussed by F. Rahmandari et al., stated that all formulations intended for application to the skin must be in the range suitable for skin, i.e., 4.0 to 6.0 [23]. It was observed that pH of the preparations kept at different environmental conditions (8 ± 2 °C, 25 ± 2 °C and 40 ± 2 °C) rose in the first week of study, but later the values were declined over the whole study, with significant variations. It was assumed that there were some acidic metabolites causing specific decline in the pH of the formulations [23]. Through statistical analysis, it was observed that the changes in the pH of the formulations were insignificant (ANOVA; *p* > 0.05) at different environmental temperatures and time intervals. The formulations showed no skin irritation and were appropriate for application to the skin.

#### 3.2.3. Homogeneity and Smear Test

The homogeneity and smear test were conducted to evaluate the uniformity of the ingredients used in the formulation. The test was conducted over a time period of four weeks at different storage conditions, as mentioned above. It was observed that the formulations (C1 and C2) were stable throughout the four weeks and no change occurred. The smear test revealed that C1 and C2 are greasy and have good moisturizing properties. Prepared creams were readily removed with tap water, indicating that they may be utilized without difficulty. The cream homogeneity test revealed that the formulations had a homogeneous composition. The colors of the prepared formulations were off white to pale yellow, as shown in Table 2, as per the organoleptic evaluation. All of the creams had a pleasant odor and a semisolid consistency. Neither of the formulations showed any phase separation (Table 3).

#### 3.2.4. Viscosity

**Figure 2:**
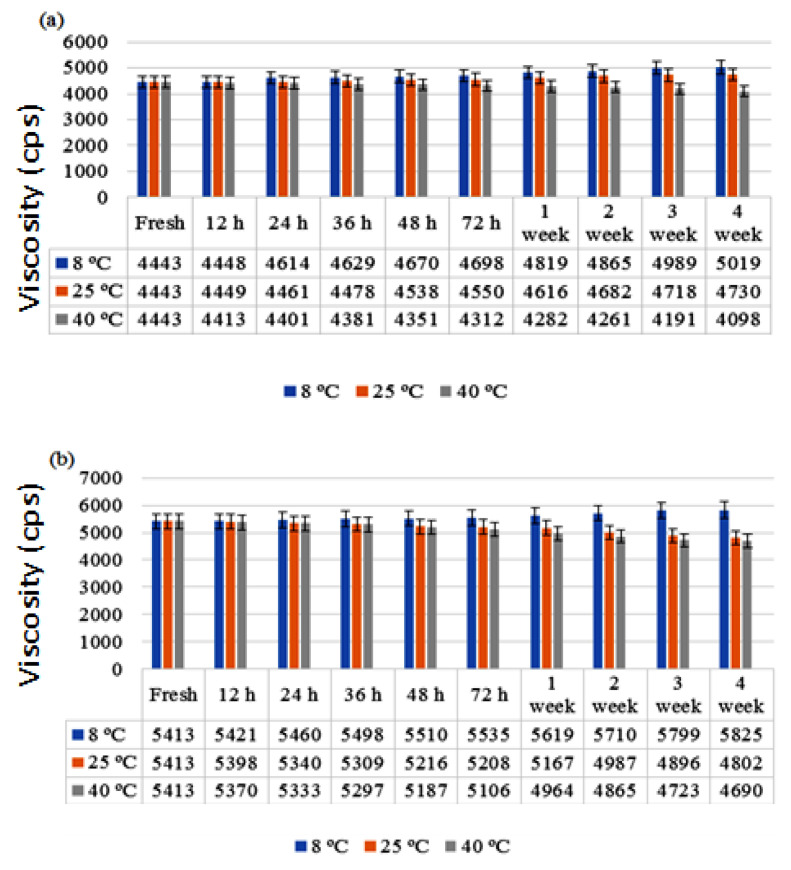
Viscosity of formulations (**a**) C1 and (**b**) C2 over 28 days.

The viscosity of any semisolid formulation usually indicates its consistency. One of the most important characteristics of topical formulations is the consistency of the semisolid mixture, as it is applied to the skin as a thin layer. It was observed that the viscosity of the cream was inversely related to the shear stress. When shear stress is increased, the viscosity reduces, showing non-Newtonian flow behavior [24]. This behavior is preferred, since it has a low resistance to flow when applied under high shear circumstances [25]. In the present study, the viscosity of the formulations was measured by using different shear stresses and different revolutions per min (6, 12, 30 and 60 rpm). It was observed that by increasing the shear stress, the viscosity of the formulations significantly declined, and vice versa. The viscosity of the developed cream formulation was measured with a Brookfield viscometer using spindle no. 2. The viscosity of creams is significant in their application, since relatively low viscosity leads to easy flow off the surface and high viscosity causes problems with spreading [26]. The viscosity of the formulations were in the order C2 > C1, but both were in the acceptable range and easily flowed off the skin (ANOVA; *p* < 0.05). It can be seen that introduction of chitosan causes a rise in the viscosity of the creams. This might be due to chitosan’s swelling nature. Surfactant molecules, micelles and oil droplets form a network as the concentration in the external phase rises. The denser the network, the higher the viscosity and the closer the gap between the dispersed phase and the yield value. As shown in Figure 2, the viscosity is inversely proportional to the temperature; the greater the temperature, the lower the viscosity, and vice versa [27].

#### 3.2.5. Spreadability

Spreadability of creams is an important parameter that shows the efficacy and extent of spreading on the skin after their application [28]. The spreadability values of the prepared creams were in the suitable range [29]. The spreadability of the prepared creams was in the order of C1 > C2 (C1-44.31 ± 1.24 g·cm/s; C2-41.34 ± 1.45 g·cm/s) (ANOVA; *p* < 0.05). The prepared cream formulations (C1 and C2) exhibited a good spreadability rate over the surface of the skin. The reason might be due to the presence of an appropriate quantity of liquid paraffin and white soft paraffin. These excipients offer lubrication and give the formulated cream good spreadability. The addition of chitosan has an insignificant effect over the spreadability of the formulated cream preparations (C1 and C2). Various factors, including low and high temperatures, affect the spreadability coefficient of a cream formulation. Indeed, at low temperatures, the viscosity of cream formulations increased, resulting in decreased spreadability. Conversely, at high temperatures, the viscosity of cream formulations decreased, resulting in high spreadability [30]. The study showed that our prepared cream formulations (C1 and C2) exhibited good and uniform spreadability (Table 3) [31].

#### 3.2.6. Drug Content

For semisolid preparations, drug content uniformity is required to ensure the homogeneity of the distributed drug throughout the formulation [32]. To ensure uniform drug distribution in the prepared cream formulations, the percentage drug content test was performed. Results showed that the percent drug contents of C1 and C2 were 95.16% and 96.12%, respectively (ANOVA; *p* < 0.05). It was also revealed that C2 has more drug entrapment in the formulation as compared to C1. This may be due to the use of chitosan as a polymer and coating agent in C2. As the concentration of polymer increased, the entrapment efficiency also increased, due to its stabilization effect [32]. The results verify that the drug content was within the approved range of 90–110% (Table 3). This demonstrates that the medication was evenly dispersed throughout the creams. As a result, the method adopted in this study appears to be suitable for the manufacturing of cream.

#### 3.2.7. Stability Studies

**Figure 3:**
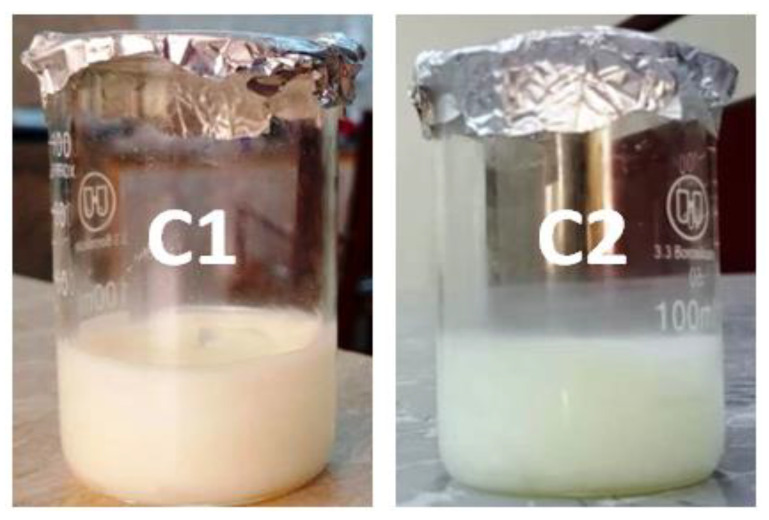
Photographs of formulations C1 and C2.

Stability studies were conducted to evaluate the physical appearance of the prepared formulation. Different parameters such as color, odor, consistency, homogeneity and phase separation were observed over the time period of 60 days (Table 4 and Figure 3). Rodrigues et al. stated that these parameters must be taken in account, as they can compromise the efficacy and presentation of the formulation, which remains either changed or unchanged [33]. In the recent study, it was ensured that the prepared cream formulations (C1 and C2) passed the test of homogeneity and no significant changes occurred in the color and odor at different temperatures (8 ± 2 °C, 25 ± 2 °C and 40 °C) in an oven for a time period of 60 days. It was also observed that no significant phase separation occurred in the specified period of time (ANOVA; *p* > 0.05). However, there was a slight variation in the pH. Y. Pakzad et al., documented that the rate of degradation of cream depends upon two parameters: pH and temperature [34]. It was concluded that the prepared formulations (C1 and C2) passed the homogeneity, pH and phase separation test and retained their integrity and stability over the time period of 60 days.

### 3.3. In Vitro Drug Release

**Figure 4:**
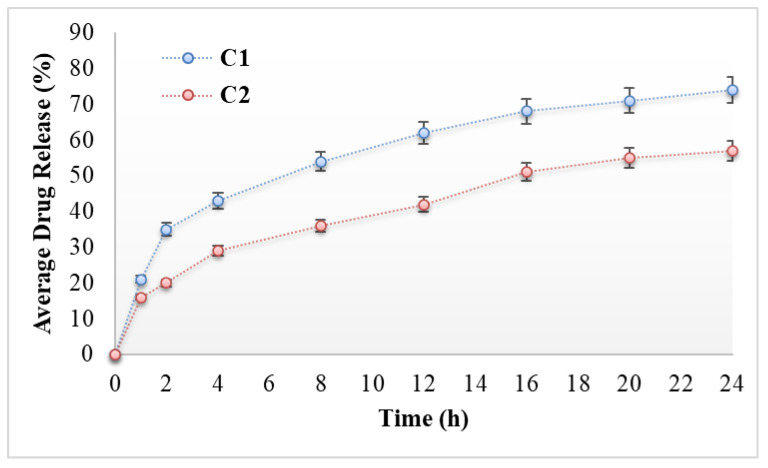
In vitro drug release profiles of C1 and C2.

It is claimed that effectiveness of any drug depends upon the drug release pattern across the cell membrane [35]. Several factors such as polymer, emulsifiers, surfactants and gelling agents greatly affect the spreadability and viscosity of a formulation, which in turn affects the drug release pattern from the topical preparation [36]. The release patterns of the drug from bacitracin-loaded cream without chitosan coating (C1) and bacitracin-loaded cream with chitosan coating (C2) are graphically presented in Figure 4. The percentage drug release of the C1 formulation was recorded as up to 74% at the end of 24 h, while the releases of the drug from the C2 formulation was found to be up to 57% for 24 h. The percentage drug release of C1 was significantly higher that of the C2 formulation (ANOVA; *p* < 0.05). The above-mentioned values show that the C2 formulation releases less of the drug as compared to C1; this is due to addition of polymer-chitosan. The presence of the gelling agent increases the integrity of the gel network, resulting in longer diffusion pathways of drug penetrating through the membrane and, hence, reduced drug release from C2 [35]. Jhaveri et al. found similar findings, claiming that release of the drug from any formulation depends upon polymer or gelling agent use, showing an inverse relationship [37].

### 3.4. Antibacterial Activity Test

**Figure 5:**
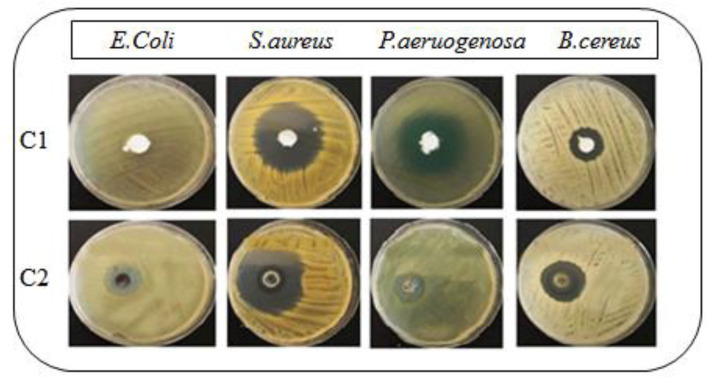
Antibacterial activity of the prepared formulations (C1 and C2) against various bacterial strains.

The kill rate of several microbes was examined using the viable cell counting technique to determine antibacterial activity. In the present study, the disk diffusion technique was used to test the susceptibility of *S. aureus* (ATCC), *E. coli* (STCC), *P. aeruginosa* (ATCC) and *B. cereus* (ATCC) against C1 (bacitracin-loaded non-chitosan-decorated cream) and C2 (bacitracin-loaded chitosan-decorated cream). All the tests were based on the guidelines of the European Committee for Antimicrobial Susceptibility Testing [38]. Chitosan’s antibacterial activity was tested against pathogenic clinical isolates and its antibiotic sensitivity was compared to a basic bacitracin-loaded non-chitosan-decorated cream. The inhibitory activity of chitosan was found to be greater against all the bacterial strains, in contrast with simple drug-loaded cream (ANOVA; *p* < 0.05). The zones of inhibition of C1 (bacitracin-loaded non-chitosan-decorated cream) were 2 ± 0.2, 28 ± 0.92, 15 ± 0.5 and 11 ± 1.25 mm, while the zones of inhibition of C2 (bacitracin-loaded chitosan-decorated cream) were 10 ± 0.6, 34 ± 1.5, 31 ± 0.76 and 21 ± 2.02 mm for different species, as shown in Table 5 and Figure 5. This was within the EUCAST-recommended quality control range [38]. Sukmark et al. explored the same phenomenon: that the antibacterial action of chitosan-decorated formulations from various sources show a larger zone of inhibition than basic antibiotics [39]. Similarly, Mauro et al. also showed that the antibacterial activity of a natural compound was enhanced in the presence of chitosan [40]. Chitosan, a cationic polymer, interacts with the anionic groups found on bacterial cell surfaces, which results in the alteration of the cell wall or outer membrane, followed by disturbances in the cytoplasmic membrane permeability and the death of the bacterial cell [41]. Chitosan also forms an impermeable layer on the surface of bacteria cells, affecting the transport of vital components into the cell [42]. The presence of polymer (chitosan) shows a synergistic effect in preventing the growth of microorganisms.

## 4. Conclusions

Topical antimicrobials have been used successfully to decrease bacterial infections in wounds for decades. Bacitracin is a broad-spectrum antibiotic with a wide range of biological intervention that may be used to prepare variety of formulations to treat inflammation, wounds and microbiological infections. In the present study, topical creams were prepared with and without chitosan coating. The prepared creams have optimum pH, viscosity, homogeneity, spreadability and drug content. The release of the drug from the cream was controlled in the presence of chitosan. The chitosan-decorated cream showed significantly larger zones of inhibition against different bacterial strains as compared to non-chitosan-decorated cream. This was attributed to the synergistic effect of chitosan, as chitosan acts as a strong antimicrobial. Based on these findings, the loading of chitosan and bacitracin into skin cream is a promising approach for further use in biomedical applications, predominantly in wound dressings.

## Figures and Tables

**Table 1:** Composition of cream (*w*/*w*).

S. No.	Ingredients	C1	C2
1	Bacitracin	1 g	1 g
2	Chitosan	-	1 g
3	Beeswax	18 g	18 g
4	Liquid paraffin	20 g	20 g
5	Cetyl alcohol	4 g	4 g
6	Distilled water	20 g	19 g
7	White soft paraffin	37 g	37 g

**Table 2:** pH value of formulations at 8 ± 2 °C, 25 ± 2 °C, 40 ± 2 °C.

Time Period	8 ± 2 °C		25 ± 2 °C		40 ± 2 °C	
	C1	C2	C1	C2	C1	C2
Fresh	5.3	5.6	5.3	5.6	5.3	5.6
12 h	5.35	5.5	5.31	5.52	5.59	5.68
24 h	5.31	5.52	5.29	5.57	5.5	5.61
36 h	5.27	5.6	5.23	5.48	5.49	5.58
48 h	5.24	5.5	5.2	5.52	5.43	5.51
72 h	5.18	5.48	5.19	5.4	5.31	5.43
1 week	5.14	5.41	5.5	5.34	5.24	5.3
2 weeks	5.09	5.3	5.09	5.2	5.12	5.35
3 weeks	4.98	5.2	4.64	4.9	5.01	5.1
4 weeks	4.72	5.21	4.53	4.7	4.91	4.9

**Table 3:** Physical appearance of prepared creams.

Parameters	C1	C2
Color	Light yellow	Off white
Phase Separation	Nil	Nil
Homogeneity	V. Good	Excellent
Consistency	V. Good	V. Good
Smear Test	Greasy	Greasy
Spreadability (g × cm/s)	44.31 ± 1.24	41.34 ± 1.45
Drug Content (%)	95.16	96.12

**Table 4:** Stability study of formulations at different temperatures for 60 days.

Parameters	Codes	Fresh	24 h	36 h	48 h	72 h	7 d	21 d	28 d	60 d
Color	C1	OW	OW	OW	OW	OW	OW	OW	OW	OW
	C2	OW	OW	OW	OW	OW	OW	OW	OW	OW
Odor	C1	-ve	-ve	-ve	-ve	-ve	-ve	-ve	-ve	-ve
	C2	-ve	-ve	-ve	-ve	-ve	-ve	-ve	-ve	-ve
Phase Separation	C1	-ve	-ve	-ve	-ve	-ve	-ve	-ve	-ve	-ve
	C2	-ve	-ve	-ve	-ve	-ve	-ve	-ve	-ve	-ve

Note: OW (off white), -ve (No change).

**Table 5:** Zone of inhibition for different bacterial strains.

Zone of Inhibition (mm)				
Strains	*E. coli*	*S. aureus*	*P. aeruginosa*	*B. cereus*
C1	2 ± 0.2	28 ± 0.92	15 ± 0.5	11 ± 1.25
C2	10 ± 0.6	34 ± 1.5	31 ± 0.76	21 ± 2.02

## Data Availability

Not applicable.
